# Structural Connectivity of the Developing Human Amygdala

**DOI:** 10.1371/journal.pone.0125170

**Published:** 2015-04-15

**Authors:** Zeynep M. Saygin, David E. Osher, Kami Koldewyn, Rebecca E. Martin, Amy Finn, Rebecca Saxe, John D.E. Gabrieli, Margaret Sheridan

**Affiliations:** 1 McGovern Institute for Brain Research, Massachusetts Institute of Technology, Cambridge, MA, United States of America; 2 Psychological and Brain Sciences, Boston University, Boston, MA, United States of America; 3 Laboratories of Cognitive Neuroscience, Boston Children’s Hospital, Boston, MA, United States of America; 4 School of Psychology, Bangor University, Gwynedd, United Kingdom; Duke-NUS Graduate Medical School, SINGAPORE

## Abstract

A large corpus of research suggests that there are changes in the manner and degree to which the amygdala supports cognitive and emotional function across development. One possible basis for these developmental differences could be the maturation of amygdalar connections with the rest of the brain. Recent functional connectivity studies support this conclusion, but the structural connectivity of the developing amygdala and its different nuclei remains largely unstudied. We examined age related changes in the DWI connectivity fingerprints of the amygdala to the rest of the brain in 166 individuals of ages 5-30. We also developed a model to predict age based on individual-subject amygdala connectivity, and identified the connections that were most predictive of age. Finally, we segmented the amygdala into its four main nucleus groups, and examined the developmental changes in connectivity for each nucleus. We observed that with age, amygdalar connectivity becomes increasingly sparse and localized. Age related changes were largely localized to the subregions of the amygdala that are implicated in social inference and contextual memory (the basal and lateral nuclei). The central nucleus’ connectivity also showed differences with age but these differences affected fewer target regions than the basal and lateral nuclei. The medial nucleus did not exhibit any age related changes. These findings demonstrate increasing specificity in the connectivity patterns of amygdalar nuclei across age.

## Introduction

The amygdala is a subcortical structure, comprised of four main nucleus groups, that is critically involved in specific emotional and learning processes in adults (e.g. [1]). Many neurodevelopmental disorders are associated with atypical amygdala function, including autism and anxiety ([2]; [3]; [4]). Despite the importance of this region, little is known about the development of the human amygdala, particularly with respect to its nuclei, which are structurally and functionally distinct.

What is known from human functional neuroimaging studies suggests that amygdala *function* (as a whole) continues to mature through adolescence (e.g. [5]; [6]; [7]). The findings concerning *structural* development of the amygdala are more mixed. Several studies report no developmental changes in amygdalar volume ([8]; [9]), but others find differential age-related changes for girls versus boys (e.g. [10]) or small volumetric increases ([11]; [12]). Given the inconsistent reports of volumetric development and the consistent observations of functional development, one possibility is that the maturing connectivity patterns of the amygdala may be the structural change that underlies functional development in this region.

Studies in non-human primates support this hypothesis and highlight the importance of the amygdala’s connectivity patterns in affecting its function in the maturing brain. Amygdala projections mature well after birth: amygdalar nuclei are connected with more regions in juvenile than adult animals, and these connections are either refined or completely eliminated through adulthood in tandem with affective and social maturation ([13]; [14]; [15]; [16]; [17]; [18]). Further, comparisons of early versus late amygdala lesions suggest that the critical role of the amygdala in social behavior and learning may change as its connections develop ([19]; [20]; [21]; [22]).

Studies in animals additionally highlight the diversity of function and connectivity of the four main nucleus groups within the amygdala (e.g. [23]; [24]; [25]). Previous developmental neuroimaging work in humans has reported on the development of the amygdala as a whole (e.g. [5]; [6]; [7]; [26]; [27]; [28]). However, it is likely that the connectivity of amygdala nuclei may offer additional information about this structure’s development in humans. Recently it was demonstrated that the nuclei could be estimated in humans, using diffusion weighted imaging (DWI; e.g. two subregions as in [29]; [30]; and four subregions as in [31]).

While these previous studies have explored amygdala connectivity in adults, it is currently unknown whether the structural connectivity of the amygdala is refined through human development. Previous DWI studies have reported broad increases in white matter volume or tract-integrity during development (e.g. [32]; [33]; [34]; [9]), but these studies were not specific to the amygdala and its projections across age. One study that addressed the question of amygdalar development reported that basolateral and centromedial nucleus groups showed less resting-state (functional) connectivity with the rest of the brain in children as compared to adults ([35]). While there is some correspondence between structural and functional connectivity, the two metrics are not identical and the degree to which functional correlations reflect anatomical connections remains unclear (e.g. [36]; [37]). Further, the basal nucleus is distinct from the lateral, both in terms of function and connectivity, as is the central from the medial nucleus (e.g. [24]; [23]); grouping these nuclei may ignore important connectivity differences between them. Thus, it remains unknown in humans i) whether the amygdala’s DWI connections are different in children versus adults; ii) whether all nucleus groups in the amygdala undergo developmental changes in DWI connectivity; and iii) which specific connections are changing across development.

Here, we measured developmental changes in the probability of connections between amygdalar nuclei and the rest of the brain using DWI probabilistic tractography (e.g. [38]; [39]; [40]; [41]; [30]; [31]). First, we tested the hypothesis that the amygdala as a whole becomes more specific in its DWI connectivity patterns with age. Next, by developing a model to predict an individual’s age based on that individual’s amygdala connectivity, we explored which brain regions’ connectivity with the amygdala drives the changes in selectivity with age. Finally, we segmented the amygdala into is four main nucleus groups using a priori defined parcellations (from [31]) and examined developmental changes in the connectivity patterns of each subregion.

## Materials and Methods

The main focus of this study is on the amygdala’s connectivity vector or fingerprints with the rest of the brain, and what components of this vector are most predictive of age in a cross-sectional sample. We divided each individual’s native anatomical brain image into a common set of 87 cortical and subcortical regions using the Desikan-Killiany Atlas ([42]) from Freesurfer; these included 2 regions: the right and left amygdala, and 85 target regions. These regions are defined separately for each individual, and retain individual anatomical variations. By establishing the correspondence of each anatomical region across subjects, this method enables us to define the connectivity of each voxel in a common currency across subjects: the connection probability of that amygdala voxel to each of the 85 other non-amygdala regions (i.e. its connectivity vector or fingerprint). We can thus derive from one set of subjects the relationship between age and connectivity (to all 85 other regions), and then apply this relationship to a new individual, and predict the age of this individual based on his/her amygdala’s diffusion-based connectivity to the 85 other parcels. We can therefore preserve individual subject DWI data without warping to a common template space, and still elucidate general principles about how the amygdala is connected to the rest of the brain, and how this may change across age. First, we compared the mean connectivity vector of the whole amygdala across participants of different ages. Next, we developed a model to predict an individual’s age based on that individual’s amygdala connectivity, and identified the amygdala connections that were most predictive of an individual’s age. Finally, we examined the developmental changes in the connectivity patterns of each of the amygdala’s subregions (defined from [31]).

### Participants

Participants were recruited from the greater Boston area and were screened for history of mental illness, MR contraindications, and known neurological abnormalities prior to scanning. After excluding participants for excessive motion (determined by visual inspection), we examined amygdala connectivity in 166 participants ranging in age from 5 to 30, composed of two groups scanned with slightly different scan parameters (see **Acquisition**). Group 1 included 64 participants (29 females; mean age ± standard deviation = 15.57 years ± 7.74) and Group 2 included 102 participants (40 females; mean age = 13.50 years ± 7.76). Participants were recruited as part of ongoing studies approved by the Committee on the Use of Humans as Experimental Subjects (COUHES) at the Massachusetts Institute of Technology. We obtained written informed consent from the guardian of each child participant, and assent from the child.

### Acquisition

Diffusion-weighted data were acquired from all participants using echo planar imaging (64 slices, voxel size 2x2x2mm, 128x128 base resolution, b-value 700s/mm^2^, diffusion weighting isotropically distributed along 60 directions for Group 1 and 30 directions for Group 2 on a 3T Siemens scanner with a 32 channel head-coil ([43]). A high-resolution (1mm^3^) 3D magnetization-prepared rapid acquisition with gradient echo (MPRAGE) scan was also acquired on all participants.

### Defining amygdala seed regions and whole-brain target regions for tractography

Automated cortical and subcortical parcellation was performed to define specific cortical and subcortical regions in each individual’s T1 scan using FreeSurfer ([44]; [45]). Automated segmentation results were reviewed for quality control, and corrected for parcellation errors if necessary using standard approaches to manual editing in FreeSurfer (by Z.S., K.K., and R.M.). They were then registered to each individual’s diffusion images, and used as the seed and target regions for fiber tracking. This resulted in 85 cortical and subcortical targets (all cortical parcellations in the 2005 FreeSurfer atlas) and 2 seed regions (the bilateral amygdala) per participant. The principal diffusion directions were calculated per voxel, and probabilistic diffusion tractography was carried out using FSL-FDT ([38]; [40]; [41]) with 25000 streamline samples in each seed voxel to create a connectivity distribution to each of the target regions, while avoiding a mask consisting of the ventricles.

### Tractographic analysis

All analyses were performed on subject-specific anatomy, rather than extrapolation from a template brain. In each participant and for each amygdala voxel, we calculated the connection probability to all of the anatomically-defined cortical and subcortical target regions. This produced a connectivity vector or fingerprint for every amygdala voxel, which we then normalized to [0,^1^], by dividing by the maximum connection probability of that voxel to all the other target regions (i.e. the rest of the brain) as previously reported ([31]). This method of normalizing probabilities per voxel accounts for lower connection probabilities in the deepest parts of the amygdala and thus allows for a comparison of relative probabilities to each target both within and across participants (e.g. [39]; [41]; [30]). As a result, we can explore the relative patterns of amygdala connectivity (i.e. connectivity vector) and can evaluate how the relative strength and sparsity of each amygdala nucleus’ connectivity patterns changes across development.

### Amygdala volume comparisons

We defined the amygdala (and all other target regions) by using Freesurfer (described above). We generated volumetric measurements by calculating the number of voxels in the binarized right and left amygdala labels in Matlab. The volumes (in mm^3^) of the right and left amygdalae were correlated across age for each hemisphere using a Pearson’s partial correlation controlling for gender and group (i.e. Group 1 with 30 direction DWI, Group 2 60 direction DWI, to account for any possible differences across study participants). Significance levels for main effects and interactions were determined as *p* < 0.05, Bonferroni corrected for the two hemispheric tests.

### Connectivity differences across age

For each amygdala voxel, we calculated the number of ipsilateral target regions that passed a connection probability threshold of 0.1 ([31]). The average ipsilateral supra-threshold connection probabilities for all voxels in the amygdala were modeled (separately for right and left amygdala) for effects of age, gender, and group using a full-factorial univariate model. We also performed the same univariate analysis using the total number of targets that were connected with the amygdala above threshold; instead of calculating the average number of targets that each amgydala voxel is connected to, we calculated the total number of targets (i.e. sum) across all amygdala voxels per individual. Prior to thresholding, we also calculated the average connection likelihood from the amygdala to each ipsilateral target region for each individual (e.g. [39]; [41]; [30]). We then averaged these connection probabilities for each amygdala and performed a Pearson’s partial correlation (controlling for gender and group) of mean connectivity values with age. All significance levels were set at *p* <. 05, Bonferroni corrected for two hemispheric tests. We also used the connection likelihoods for each target region separately for the analyses below.

### Specific connectivity changes with age

We used a machine-learning approach (Support Vector Machine, SVM) to model the relationship between age and amygdala connectivity, because such approaches are robust to noise, flexible even with high-dimensional data ([46]; [47]) and informative for determining which features (amygdala connectivity to each ipsilateral target) of a dataset are most relevant for modeling the response variable (in this case, age). With an SVM, each sample is treated as a point in *n*-dimensional space, where *n* is the number of features (connectivity targets); the support vector regression (SVR) then finds the regression line that best fits the points in this hyperspace. Here, the connectivity data of ipsilateral targets to the right and left amygdalae were used as features to model chronological age with a nested leave-one-subject-out cross-validation approach (LOOCV). This was performed using in-house MATLAB (R2011b; The Mathworks, Natick, MA) code and LibSVM toolbox (http://www.csie.ntu.edu.tw/~cjlin/libsvm/).

The model was built on age and connectivity data concatenated across all but one participant, and tested using the remaining participant’s connectivity data. This was performed iteratively for all participants. As is common for machine-learning approaches, we used a grid-search nested cross-validation routine in order to improve model fits while avoiding over-fitting. Within each loop of the LOOCV, optimal model parameters and features were discovered via nested cross-validation during which the remaining subjects were randomly partitioned into three groups and independently fit using ν-support vector regression with a Gaussian radial basis function kernel, varying ν (0.2, 0.5, 0.8), γ (2^–4:1^), and c (2^–1:3^) parameters. Features were selected by computing the Pearson correlation coefficient separately for each nested partition, and selecting the 30 highest correlations. The model applied to the left-out subject in the outer LOOCV loop was derived from the most accurate fitting model and consensus features from the independent group of nested partitions. The relative weights of these features for predicting age from connectivity in the final model were generated by fitting a final model on all subjects using the most common parameters (ν = 0.8, γ = 2^–4^, c = 2^3^), and consensus features (those features that appeared in all LOOCV loops). Since our feature selection routine was univariate, it is not influenced by issues such as multicollinearity or redundancy, and thus reflects the features that are changing most with age. The final model weights, on the other hand, may be influenced by these factors; however, the reported correlations compliment these weights and the ordering closely follows the rank ordering of the weights.

### Timing of specific connectivity changes in amygdala subregions

We then used a probabilistic atlas to explore the changes in connectivity with specific subregions of the amygdala. The atlas was derived from a previous study ([31]) that used the differential connectivity patterns of four main nuclei (based on known connectivity patterns from animal work) to segment the amygdala in adults, which was validated through high-resolution anatomical imaging. We used the probabilistic atlas instead of individually-defined nuclei because a) we wanted to explore the connections of the amygdala and avoid the circularity of using the connections to define the nuclei; and b) we do not expect the most probable voxels in each nucleus (i.e. the centers of each nucleus) to drastically change in location across postnatal development. Importantly, this method employs native-space anatomy to register amygdalae of different participants, rather than normalization to a template, which may add unwanted warping or misalignment of data. We used this approach here since it preserves individual amygdala anatomy, which is important for studying development. In order to test for volumetric differences across age in the probabilistic nuclei, the volume (in mm^3^) of the right and left subregions were correlated with age, using partial correlations in the same manner as for the connectivity-age correlations. We also checked the patterns of connectivity for each nucleus between children and adults; in [31], each nucleus was defined using a unique and characteristic connectivity vector. We extracted these connectivity vectors for the voxels falling within the boundary of each amygdala subregion, calculated a mean connectivity vector for children (below the age of 18) and a separate vector for adults (ages 18–30), and correlated these vectors between children and adults. We also performed a paired t-test to explore any differences in overall connection strengths for each nucleus. These analyses suggested that the core connections for each subregion (except the medial nucleus) were already adult-like in children, but higher. We therefore examined the age-related differences in these subregions for the targets that were chosen from the model for the whole amygdala. The connectivity values for each of these targets were extracted for each amygdala nucleus and collapsed across hemispheres. For each nucleus, we correlated these connectivity values with age using a Pearson’s correlation and assessed significance at *p* < 0.05 corrected for 13 tests. Fisher Z-tests were then used to compare correlation strengths per target for each nucleus.

## Results

### Volume and Connectivity differences across age

**Fig 1:**
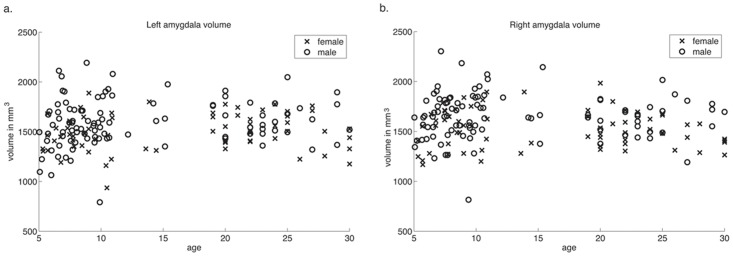
Amygdala volume across age. Amygdala volume is plotted by age. Volume does not correlate with age in either the **a.** left or **b.** right amygdala (left: *r* = 0.13, *p* = 0.11; right: *r* = –0.02 *p* = 0.80).

Because we hypothesized that connectivity changes may be the structural substrate for functional changes in the amygdala, and given the prior limited literature on volumetric development, we first compared amygdala volumes across age. We found no age-related changes in amygdala volume, controlling for gender and group (left: *r* = 0.13, *p* = 0.11; right: *r* = –0.02 *p* = 0.80; Fig 1a and 1b).

**Fig 2:**
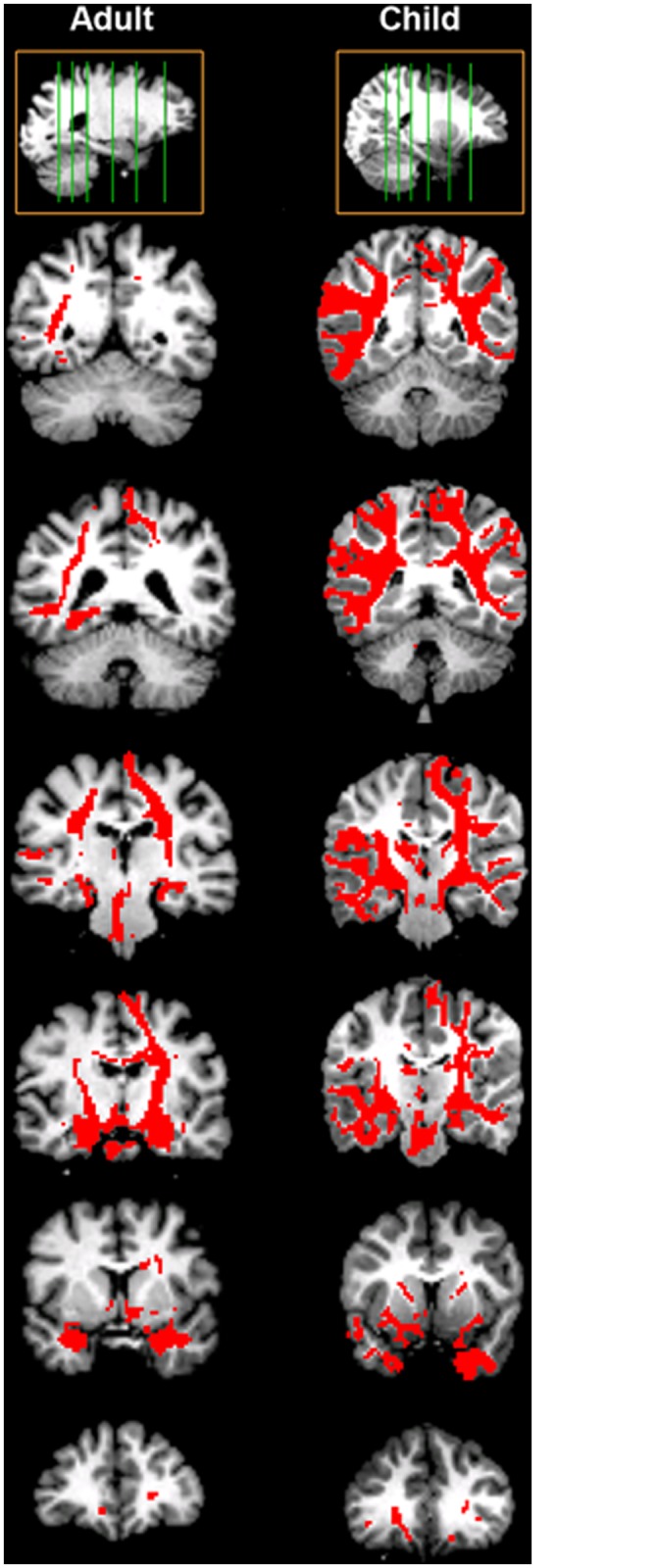
Probabilistic tracts of amygdala connectivity for two example subjects. Probability maps are thresholded at 0.1 of maximum connection probability for each subject and overlaid on the same subject’s low-b diffusion image. These depict all possible tracts that the tractography algorithm used to connect the left and right amygdalae with all other target regions (i.e. the rest of the brain). Top images are the sagittal sections showing the slice locations for the coronal sections below for the adult subject (left column) and child subject (right column). Coronal slices progress posterior to anteriorly through each subject’s brain. Each slice corresponds to a similar anatomical location in both subjects but the match is not perfect due to differences in head orientation and anatomy. The adult and child subject show the same major pathways that are reconstructed by the tractography algorithm. However, the example child’s map illustrates more widespread connections than the adult participant’s map; e.g. to parietal and temporal regions; see 4^th^ and 5^th^ row from the bottom.

**Fig 3:**
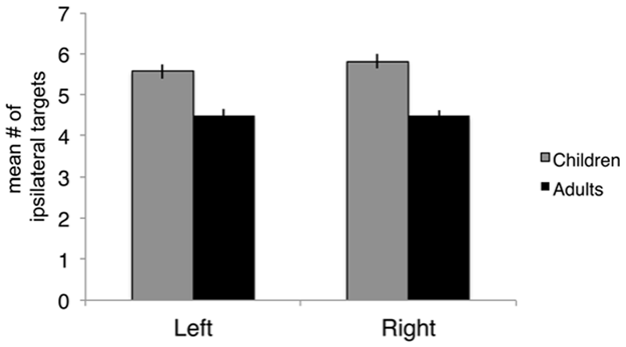
Mean number of ipsilateral target regions that the amygdala connects with in children and adults. Each amygdala voxel’s connectivity vector to all other target regions is thresholded at 0.1, binarized, and averaged across the amygdala per individual. In children, the amygdala showed connectivity (above 0.1) to a greater number of target regions on average than adults in both left (C: 5.57±0.18, A: 4.50± 0.16) and right (C: 5.81± 0.18, A: 4.48±0.13).

Qualitative comparisons of the probabilistic tractography maps from the bilateral amygdalae to target regions across the brain revealed that similar pathways for both the child and the adult example subject. However, the child subject’s probability map showed more widespread connections than the adult participant’s map (Fig 2). For example, while both the adult and child maps showed connectivity to parietal and temporal regions, the child map revealed more traces arriving at these target regions (Fig 2). We calculated the average connection likelihood from the amygdala to each target region (i.e. % traces arriving at each target region), and binarized the data for each target region, thus determining the average number of target regions that any given amygdala voxel was connected to (above threshold) in children and adults. We found that both right and left amygdalae were connected with a greater number of target regions in children than adults (Fig 3). When we treated age as a continuous variable, we found that the correlation of mean number of targets with age was significant (partial correlation controlling for gender and group; left: *r* = –0.43, *p* = 1.41x10^-8^; right: *r* = –0.50, *p* = 1.59x10^-11^). Thus, on average, voxels in both right and left amygdalae were connected (above threshold) to more regions across the brain in children than in adults.

We also calculated the total number of ipsilateral targets that all amygdala voxels connect to at the threshold of 0.1 for each individual. This differs from the analysis above, where we computed the mean number of targets across amygdala voxels; rather, here we computed the total sum of unique targets for each subject’s amygdalae as a whole. We again found significant differences between children and adults, where children showed connectivity to a greater number of targets than adults for the left amygdala (adult mean: 20.29±0.53; child mean: 22.17±0.43; t-test of children vs. adults: p = 7.10 x10^-3^; t = 2.73) and for the right amygdala (adult mean: 20.70±0.54; child mean: 22.80±0.36; t-test of children vs. adults: p = 9.81x10^-4^; t = 3.34).

**Fig 4:**
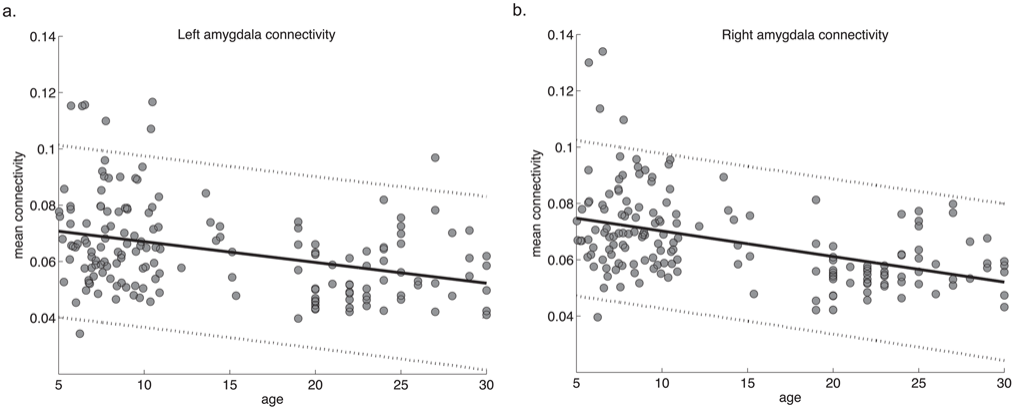
Mean connectivity values with age. Mean connectivity values per participant are plotted by age and were significantly correlated for the **a.** left and **b.** right amygdala (left: *r* = –0.43, *p* = 1.41x10^-8^; right: *r* = –0.50, *p* = 1.59x10^-11^). Dashed lines indicate 95% confidence intervals.

Next, we determined the average connection strength across all targets and found that mean connectivity also significantly decreased with age in both the left (*r* = –0.45, *p* = 1.26x10^-9^; Fig 4a) and right amygdala (*r* = –0.53, *p* = 2.59x10^-13^; Fig 4b), controlling for gender and group. We also computed the average connection strength of only suprathreshold targets (i.e. mean connectivity after applying a connectivity threshold of 0.1). This measure also significantly correlated with age (left amygdala *r* = –0.38, *p* = 5.91x10^-7^; right: *r* = –0.31, *p* = 3.61x10^-5^; S1 Fig).

### Specific connectivity changes with age

In order to further understand the differences across age in connectivity patterns, we used a leave-one-out cross-validation approach to build a model that would best predict each participant’s age based on the structural connectivity patterns of the amygdala. Because the model is built using only an optimal number of features (targets that the amygdala is connected with), the features used by the best model would reveal the specific regions that changed most with age. Each loop of the cross-validation model determined at least 30 regions whose connectivity with the ipsilateral amygdala was significantly predictive of age; 26 regions were consistently chosen by the model across all cross-validation loops. Out of these consensus features, 21 survived Bonferroni correction for multiple comparisons (*p* < 0.05/85 cross-validation loops; Table 1).

**Table 1:** Magnitude of developmental changes.

Regions decreasing with age	Weight	*r*	*p*
**Parietal**			
L inferior parietal	-111.77	-0.30	9.28x10^-5^
R inferior parietal	-107.92	-0.35	3.13x10^-6^
R precuneus	-104.78	-0.28	1.99 x10^-4^
R supramarginal	-104.08	-0.31	4.00x10^-5^
L supramarginal	-99.22	-0.31	4.79x10^-5^
R superior parietal	-97.33	-0.31	5.79x10^-5^
**Occipitotemporal**			
R bank of STS	-107.56	-0.33	8.74x10^-6^
R middle temporal	-96.37	-0.34	8.56x10^-6^
L entorhinal	-79.58	-0.31	3.59x10^-5^
R entorhinal	-67.62	-0.30	9.37x10^-5^
**Basal ganglia/Subcortical**			
L pallidum	-124.23	-0.39	2.41x10^-7^
R pallidum	-94.43	-0.40	8.26x10^-8^
L thalamus	-81.19	-0.309	5.02x10^-5^
L putamen	-70.16	-0.31	5.29x10^-5^
R putamen	-64.75	-0.29	1.40x10^-4^
L ventral diencephalon	-100.74	-0.49	1.46x10^-11^
R ventral diencephalon	-57.89	-0.40	1.21x10^-7^
**Regions increasing with age**	**Weight**	***r***	***p***
**Medial temporal**			
L hippocampus	82.89	0.44	3.36x10^-9^
R hippocampus	103.85	0.43	6.14x10^-9^
L parahippocampus	133.10	0.47	1.91x10^-10^
R parahippocampus	146.32	0.51	2.05x10^-12^

Regions used in the final SVR model, correlations with age, and the relative weights of each region in the model.

**Fig 5:**
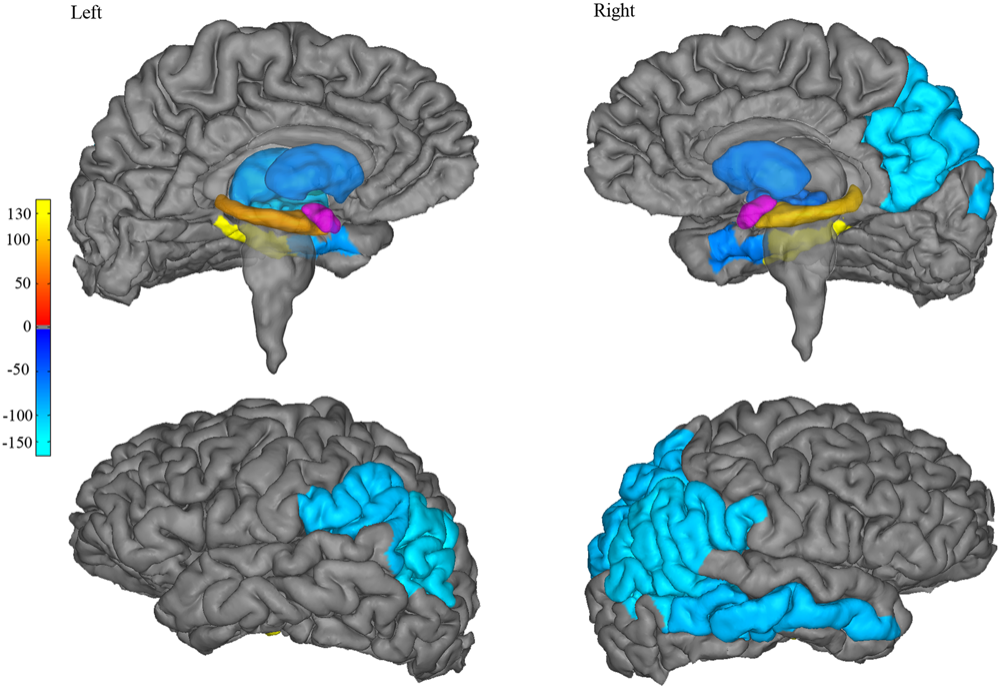
Changes in connectivity with age. The weight, or contribution, of each target region in the final model of age by amygdala connectivity, are displayed. Negative weights represent decreasing connectivity with age, and range from dark-blue (corresponding to lower absolute weights in the model) to light-blue (higher absolute weights). Positive weights (increasing connectivity with age) are illustrated by the red- (low weights) to-yellow (high weights) colors. Right and left amygdalae are depicted in purple.

The optimal features used to model age with connectivity were specific to particular occipitotemporal, parietal, basal ganglia, and subcortical regions (Table 1). Most of the regions used in the final model revealed decreasing amygdala connectivity with age. Each of the regions had a different contribution, or weight, to the predictions of age by connectivity. Parietal regions contributed the most to the overall decrease in connectivity with age, followed by the bilateral pallidum and putamen, middle temporal cortex and the bank of the superior temporal sulcus (STS). These regions were primarily right-lateralized (Fig 5). In contrast, only four regions showed increasing connectivity with age, or positive weights in the model: the bilateral hippocampus and parahippocampal cortices.

### Timing of specific connectivity changes in amygdala subregions

We explored the extent to which the subregions or nuclei of the amygdala contributed to the overall changes in connectivity observed across the amgydala as a whole. A probabilistic atlas of amygdala subregions, originally derived from a novel method of tractographic segmentation ([31]), was overlaid on each participant’s native diffusion-space amygdala. To eliminate the possibility that any connectivity differences across age were due to volumetric differences across age, we calculated the volume of each subregion per individual and performed partial correlations with age controlled for gender and study group. No significant correlations were found between the probabilistic nuclei’s volumes and age (Table 2).

**Table 2:** Volumetric measurements in the probabilistic nuclei and correlations with age (controlling for gender and study group).

Nuclei	Left		Right	
	*r*	*p*	*r*	*p*
**Basal**	0.11	0.15	-0.03	0.74
**Lateral**	0.02	0.83	-0.03	0.72
**Central**	-0.12	0.14	-0.02	0.84
**Medial**	0.06	0.48	-0.02	0.82

**Fig 6:**
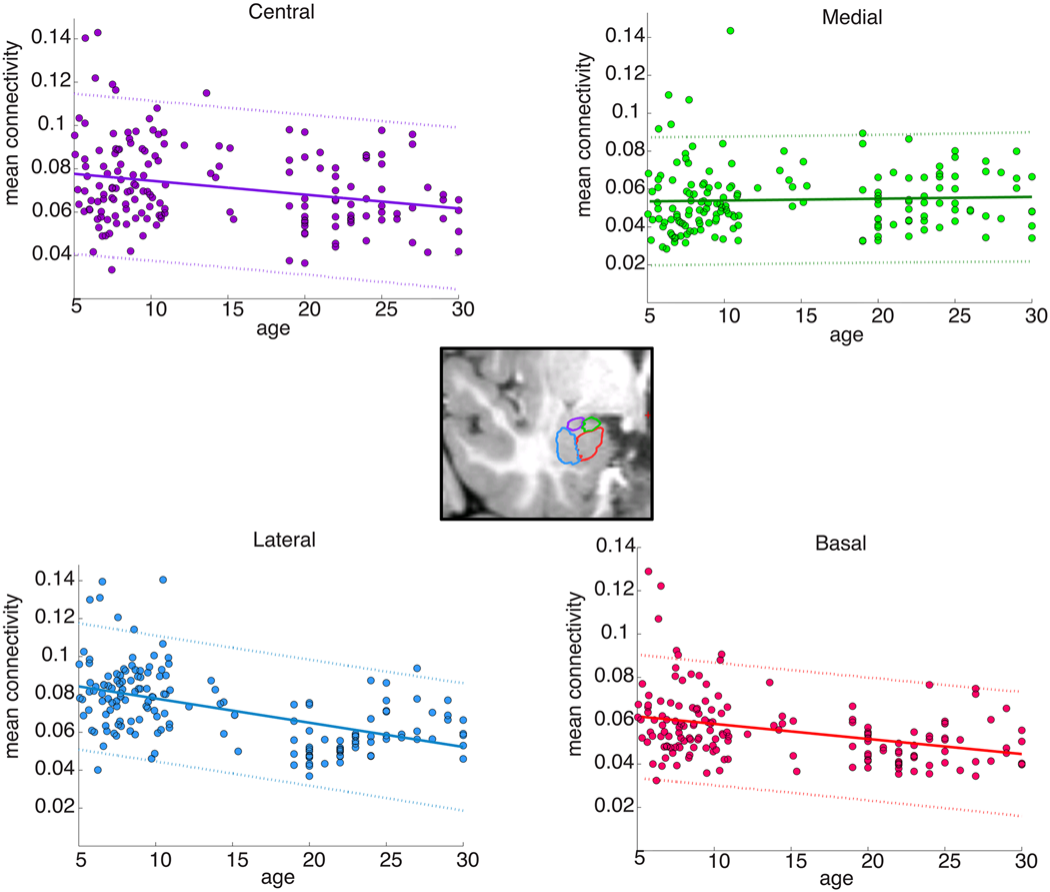
Correlations of age with mean connectivity for the four amygdala subregions. A probabilistic atlas of four amygdala subregions (illustrated in the center; coronal slice) was used to extract mean connectivity values from each subregion bilaterally per subject and plotted by age. While connectivity with the basal, lateral, and central subregions were significantly correlated with age, connectivity with the medial amygdala showed no significant change with age.

Given this lack of change in volume across age, potential developmental changes in connectivity for each subregion were then explored. Because connectivity values to each target region were calculated per voxel of the amygdala, the mean connectivity values for ipsilateral targets from voxels within each probabilistic nucleus were extracted and collapsed across hemisphere. These were then correlated with age while controlling for gender and study group (Fig 6). The basal and lateral amygdala had a clear relationship with age (basal: *r* = –0.36, *p* = 2.78x10^-6^; lateral: *r* = –0.55, *p* = 3.83x10^-14^), as did the central (*r* = –0.32, *p* = 3.41x10^-5^). The medial amygdala had mean connectivity values that were relatively stable across ages 5–30 (*r* = –7.31x10^-3^, *p* = 0.093).

We next compared the patterns of connectivity for each nucleus between children and adults. We extracted the connectivity vectors for each nucleus and compared the average vector for children to the average vector for adults. For each nucleus, the child and adult connectivity patterns were highly correlated (all r>0.9), suggesting that the relative distribution of connection strengths was very similar between children and adults. However, these connections were higher for children than for adults for the lateral (*p* = 3.20x10^-3^; t = 3.04), basal (*p* = 0.034; t = 2.16), and central nuclei (*p* = 0.029; t = 2.23), but not different for the medial nucleus. Thus, the core connections for each subregion (except the medial nucleus) were already adult-like in children, but higher. We therefore proceeded to examine the age-related differences in these subregions for the targets that were chosen from the model for the whole amygdala.

We tested for any differences between the individual amygdala subregions in how their connectivity patterns changed with age for each of the individual target regions—are such changes during development unique to particular nuclei, or do all regions of the amygdala show similar developmental changes in connectivity? As the medial nucleus’ connectivity patterns were not changing with age, we focused these analyses on the basal, lateral, and central nuclei. Each subregion’s mean connectivity to the consensus features for the whole amygdala (collapsed across hemisphere) were correlated with age (Table 3). The lateral subregion’s connectivity with all of the targets except the bank of STS changed significantly with age (Bonferroni corrected at *p* < 0.05/13 regions). Except for the hippocampal and parahippocampal regions, all targets were decreasing in connectivity with age. Similarly, all but three of the basal subregion’s targets (entorhinal, precuneus, and superior parietal) had significant changes with age (*p* < 0.05/13). In contrast, only 8 of the central amygdala’s targets showed a significant correlation with age.

**Table 3:** Correlation of connectivity to target regions with age per nucleus.

Regions	Basal		Lateral		Central	
	*r*	*p*	*r*	*p*	*r*	*p*
**Inferior parietal**	-0.32	3.47x10^-05^	-0.38	4.84x10^-07^	-0.33	1.48x10^-05^
**Precuneus**	-0.18	1.87x10^-02^	-0.37	1.22x10^-06^	-0.27	4.50x10^-04^
**Supramarginal**	-0.27	3.69x10^-04^	-0.36	1.90x10^-06^	-0.32	3.40x10^-05^
**Superior parietal**	-0.19	1.37x10^-02^	-0.34	8.34x10^-06^	-0.28	3.28x10^-04^
**Bank of STS**	-0.23	3.55x10^-03^	-0.21	7.05x10^-03^	-0.29	1.32x10^-04^
**Middle temporal**	-0.33	1.31x10^-05^	-0.23	2.75x10^-03^	-0.28	2.37x10^-04^
**Entorhinal**	-0.02	7.92x10^-01^	-0.36	1.39x10^-06^	-0.45	1.07x10^-09^
**Pallidum**	-0.37	8.44x10^-07^	-0.53	2.10x10^-13^	-0.12	1.18x10^-01^
**Putamen**	-0.32	3.34x10^-05^	-0.37	7.10x10^-07^	-0.15	4.89x10^-02^
**Thalamus**	-0.30	8.06x10^-05^	-0.38	4.37x10^-07^	-0.01	9.25x10^-01^
**Ventral diencephalon**	-0.49	4.17x10^-11^	-0.58	2.17x10^-16^	0.04	5.76x10^-01^
**Hippocampus**	0.46	8.97x10^-10^	0.48	3.89x10^-11^	0.10	1.83x10^-01^
**Parahippocampus**	0.48	1.09x10^-10^	0.48	4.15x10^-11^	0.43	8.23x10^-09^

To directly compare the amygdala subregions, we employed a Fisher’s Z test for correlation coefficients of connectivity by age per target region. The basal and lateral nuclei differed only in their connectivity strength with entorhinal cortex (*p* = 1.11x10^-3^). Both the basal and lateral nuclei showed greater change with age than the central nucleus, for connectivity with the hippocampus (BvC: *p* = 4.72x10^-4^; LvC: *p* = 1.29x10^-4^), pallidum (BvC: *p* = 1.47x10^-2^; LvC: *p* = 2.41x10^-5^), putamen (*p* = 3.14x10^-2^), thalamus (BvC: *p* = 5.83x10^-3^; LvC: *p* = 3.89x10^-4^), and ventral DC (BvC: *p* = 2.13x10^-7^; LvC: *p* = 1.62x10^-10^). In sum, although all three nuclei showed decreasing cortical connectivity with age, changes in the central nucleus’ connectivity affected fewer targets than the basal and lateral, and only the basal and lateral nuclei showed decreasing subcortical connectivity; these two nuclei were also quite similar to one another in correlation strengths of connectivity with age. Further, the basal and lateral subregions had significantly greater increases in connectivity with age for hippocampal targets as compared to the central subregion, and stronger decreases in connectivity with age for basal ganglia targets.

## Discussion

We investigated the developmental changes in the structural connectivity of the human amygdala with the rest of the brain, in over 150 subjects aged 5 to 30 years. We found that the amygdala is connected with a broad range of cortical and subcortical regions in children and becomes increasingly sparser and more targeted in its connections with age. These age-related changes were not general to all targets of the brain, but rather were specific to a subset of the amygdala’s connectivity pattern; further, these changes were observed only in the basal, lateral, and central nuclei of the amygdala, but not the medial nucleus.

Our analyses revealed the target brain regions for which connectivity with the amygdala was most predictive of age. Younger ages were predicted by higher amygdala connectivity to certain occipitotemporal and subcortical/basal ganglia regions (higher relative to all other brain regions) while higher connectivity to the parahippocampus and hippocampus predicted older ages. Because we employed a data-driven whole-brain connectivity fingerprint approach (instead of pre-selecting target regions for amygdala tractography), we also discovered unexpected predictors of age, such as the parietal cortices. Even though the parietal cortices are not commonly considered as part of the ‘affective’ or limbic network, tracer studies have revealed direct connections with the amygdala in other primates ([48]; [49]; [50]). Our results suggest that these connections can be detected with tractography, and further demonstrate the benefits of using DWI fingerprints, combined with machine-learning approaches, to explore connectivity patterns in the human brain. Future studies may validate these results more directly through more invasive techniques in other animals involving tracer injections and/or lesions.

Most of the predictive connections decreased with age (e.g. occipitotemporal regions), which may be evidence of increasing sparsity as connections are pruned or eliminated during development (e.g. [13]; [51]; [52]; [53]). Prior research in macaques is consistent with increasingly sparse amygdala connectivity with age; in addition to already adult-like projections between higher-order temporal regions and the amygdala, projections from lower-visual areas to the amygdala also exist in infant monkeys, and are later eliminated when the higher-order cortices are fully mature ([13]; [14]). It is unclear whether the occipitotemporal regions that we find here are homologous to the visual regions reported in macaques. Further, we did not perform functional imaging to localize visual or any other functionally selective regions in our participants. Thus, while our findings are consistent with evidence from macaque studies in terms of decreasing connectivity, it remains a task for future work to identify homologous regions and test whether the increasing sparsity of amygdalar connectivity is indeed causally related to increasing specialization and functional maturation of the cortex.

The age-related increases in the sparsity of DWI connectivity that we find here are complementary to the age-related increases in the functional coupling of the amygdala with the rest of the brain as measured with functional connectivity ([35]), and we identify similar brain regions that show age-dependent connectivity changes. One notable exception is the lack of developmental differences in fronto-amygdalar connectivity in our study; it is possible that the connectivity changes are at a finer-grain than what is captured by our current anatomical atlas, and that a finer parcellation of the frontal cortex would reveal these connectivity changes. This previous study also showed that the basolateral and centromedial nucleus groups were both increasing in their functional connectivity with the rest of the brain, and that the target networks of the two nucleus groups were yet undifferentiated in children ([35]). The present study also shows increasing sparsity and specialization in nucleic connectivity with age. We further show, at a finer grain than basolateral versus centromedial, that the basal, lateral, and central nuclei change in their DWI connections with age, but that the medial nucleus does not. While there is generally good correspondence between structural and functional connectivity ([54]; [36]; [55]; [56]), the two measures are not identical and functional correlations may not always reflect monosynaptic anatomical connections ([57]; [37]; [58]). The two methods have complementary strengths and limitations and it will be important to collect convergent evidence from both types of connectivity to best investigate the development of amygdala connectivity patterns, and test how pruning is linked to functional connectivity.

We also observed two brain regions whose amygdalar connectivity increased with age, becoming some of the most strongly connected regions in adults: the parahippocampal cortex and hippocampus. While amygdala connectivity overall became sparser with age, these target regions were more connected with the amygdala in adults than in children. Given the functional role of these regions in contextual processing and memory formation, these findings may imply an increasing role for the amygdala in integrating emotional content during contextual processing and memory encoding ([59]; [60]; [61]; [62].

Amygdala nuclei each have different functions (e.g. [24]; [23]) and the findings here, consistent with studies of non-human primates (e.g. [13];[63]; [64]), suggest developmental differences between these nuclei in both connectivity patterns and how those patterns change across development. The present findings revealed that in humans, the medial nucleus does not increase or decrease in its connections with the rest of the brain across age. The central nucleus exhibited some changes in its connectivity patterns with age but the connectivity of the basal and lateral nuclei showed the strongest correlations with age, and best reflected the changes seen at the level of the whole amygdala. This suggests that the developmental changes observed at the level of the whole amygdala are principally the result of development of these nuclei, and is in line with the lateral and basal nuclei’s integral role in emotional and social learning and integration of visual stimuli with value ([25]; [65]). In contrast, the central nucleus has an important role as an output nucleus for motor responses to conditioned stimuli, and the medial nucleus is involved in olfactory/gustatory responses ([1]; [66]; [67];[68]).

Developmental changes in the connectivity patterns of the basal, lateral, and central nuclei with the parietal and occipitotemporal cortices were similar to one another, in contrast to the development of connectivity patterns for these nuclei to the hippocampus and basal ganglia. Unlike the central nucleus, connectivity between basal and lateral nuclei and the basal ganglia decreased with age while their connectivity with the hippocampus increased. This pattern may reflect separation of the basal and lateral nuclei from striatal circuitry and their assimilation with medial temporal circuitry, and may be functionally relevant in forming the mature network that subserves adult emotional memory (e.g. [69]).

Previous DWI studies have reported that in general, developmental white matter changes include increases in fractional anisotropy (FA) and decreases in mean diffusivity (MD) over large areas, both of which indicate increased white matter organization (e.g. [33]; [34]; [9]). Some of these changes occur well into adulthood in tracts such as the uncinate fasciculus, which connects medial temporal lobe (MTL) structures with frontal targets ([9]). The present study focuses on a particular structure of the MTL and extends these previous results to specific amygdala-target connections. Further, the present results are unlikely confounded by previously reported developmental trajectories of white matter (e.g. increased FA) because the metric of connectivity used here reflects the pattern and sparsity of connectivity. Rather, our results expand previous findings because both connectivity and FA measures suggest increasing white matter integrity and specialization through development. One potential limitation of this study is the use of two different acquisition types for DWI (30 and 60-direction DWI). We control for this potential confound by using individual-subject data, normalizing connectivity vectors within each voxel, and including this variable as a regressor in all of our analyses. And although previous work reports that the DWI tractography method we use here yields the best solutions for DWI data with at least 30 diffusion-weighted directions ([40]), we cannot eliminate this variable as a limitation for this study.

We show the feasibility of using DWI to explore the connectivity fingerprints of the amygdala over development. Unlike tracer studies, which are the current gold-standard for measuring connectivity, non-invasive methods such as DWI are indirect and have their limitations, such as the inability to distinguish between afferent and efferent connections, possibility of false positive or polysynaptic connections due to noise, or the problem of crossing-fibers ([70]; [71]; [72]; [73]). The connectivity patterns that we study here are anatomically plausible but tracer and lesion studies will continue to be an important guide for understanding the developing amygdala and the implications of the developing connectivity patterns for functional maturation. However, non-invasive methods are the only alternative for studying the development of human brain anatomy in both health and illness. The present findings demonstrate the dynamic developmental trajectory of connectivity with the amygdala, and discern the specific anatomical targets whose connectivity changes the most.

## Conclusions

We found that the amygdala becomes more selective in its DWI connections with the rest of the brain across development, and that the basal and lateral nuclei showed the strongest age-related changes. Exploring the amygdala’s relative connectivity patterns across development, especially with respect to its functionally distinct nuclei, can be informative about specific connections, which in turn can suggest hypotheses about the bases of functional maturation of the amygdala.

## Supporting Information

S1 Fig Correlations of age with mean connectivity to only suprathreshold targets. The average connection strength was significantly correlated with age after applying the threshold of 0.1 (left amygdala *r* = –0.38, *p* = 5.91x10^-7^; right: *r* = –0.31, *p* = 3.61x10^-5^). Dashed lines indicate 95% confidence intervals.
(TIF)
