# Supplementary figures and images for: Structural Connectivity of the Developing Human Amygdala
Source: PLoS One. 2015 Apr 15;10(4):e0125170. doi: 10.1371/journal.pone.0125170 (PMC4398350; doi:10.1371/journal.pone.0125170)

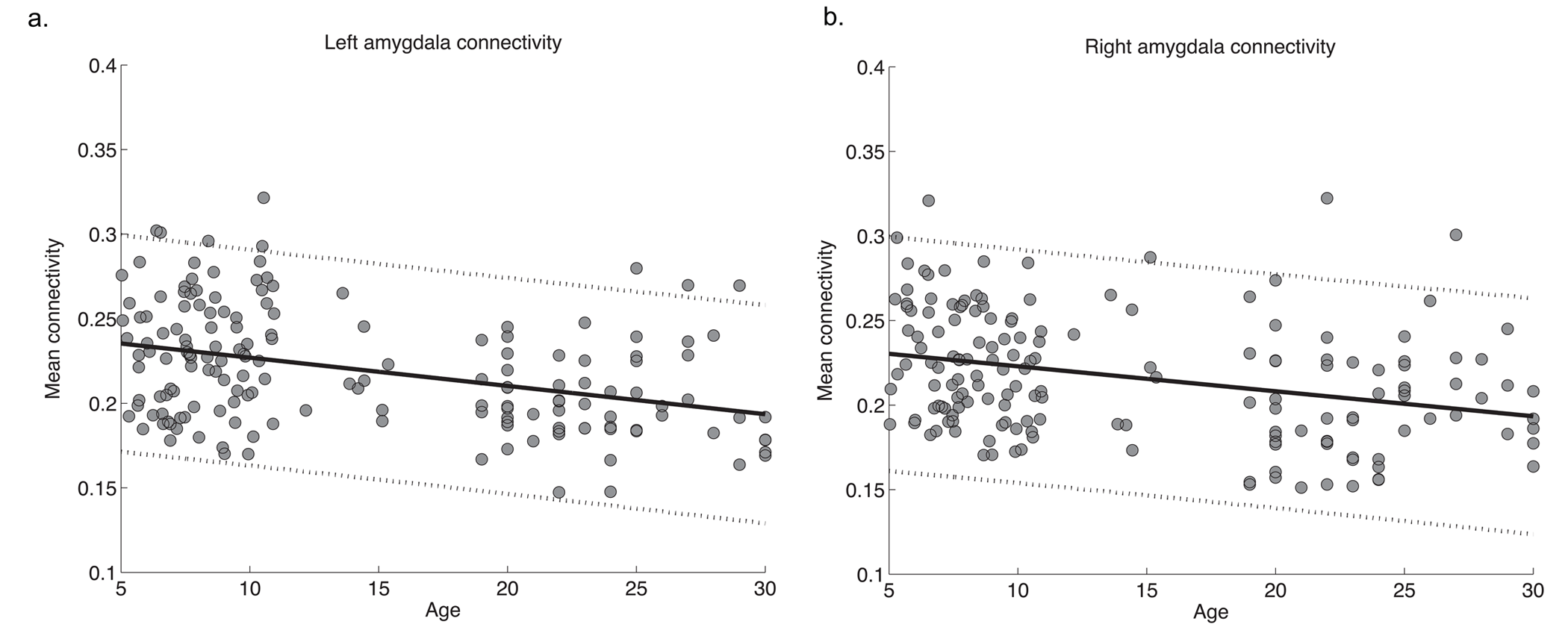

Supplement: S1 Fig — The average connection strength was significantly correlated with age after applying the threshold of 0.1 (left amygdala r = –0.38, p = 5.91x10^-7^; right: r = –0.31, p = 3.61x10^-5^). Dashed lines indicate 95% confidence intervals. (TIF) [file pone.0125170.s001.tif]
